# Reassessment of Non-Monosynaptic Excitation from the Motor Cortex to Motoneurons in Single Motor Units of the Human Biceps Brachii

**DOI:** 10.3389/fnhum.2017.00019

**Published:** 2017-01-30

**Authors:** Tsuyoshi Nakajima, Toshiki Tazoe, Masanori Sakamoto, Takashi Endoh, Satoshi Shibuya, Leonardo A. Elias, Rinaldo A. Mezzarane, Tomoyoshi Komiyama, Yukari Ohki

**Affiliations:** ^1^Division of Sports and Health Science, Chiba UniversityChiba, Japan; ^2^Department of Integrative Physiology, Kyorin University School of MedicineTokyo, Japan; ^3^Department of Physical Medicine and Rehabilitation, Center for the Neural Basis of Cognition, Systems Neuroscience Institute, University of PittsburghPittsburgh, PA, USA; ^4^Faculty of Education, Department of Physical Education, Kumamoto UniversityKumamoto, Japan; ^5^Faculty of Child Development and Education, Uekusa Gakuen UniversityChiba, Japan; ^6^Neural Engineering Research Laboratory, Department of Biomedical Engineering, School of Electrical and Computer Engineering, University of CampinasCampinas, Brazil; ^7^Biomedical Engineering Laboratory, Escola Politecnica, Departamento de Engenharia de Telecomunicações e Controle (PTC), University of Sao PauloSao Paulo, Brazil; ^8^Laboratory of Signal Processing and Motor Control, College of Physical Education, University of BrasíliaBrasília, Brazil

**Keywords:** pyramidal tract, transcranial electrical stimulation (TES), transcranial magnetic stimulation (TMS), primary motor cortex (M1), motor unit, humans

## Abstract

Corticospinal excitation is mediated by polysynaptic pathways in several vertebrates, including dexterous monkeys. However, indirect non-monosynaptic excitation has not been clearly observed following transcranial electrical stimulation (TES) or cervicomedullary stimulation (CMS) in humans. The present study evaluated indirect motor pathways in normal human subjects by recording the activities of single motor units (MUs) in the biceps brachii (BB) muscle. The pyramidal tract was stimulated with weak TES, CMS, and transcranial magnetic stimulation (TMS) contralateral to the recording side. During tasks involving weak co-contraction of the BB and hand muscles, all stimulation methods activated MUs with short latencies. Peristimulus time histograms (PSTHs) showed that responses with similar durations were induced by TES (1.9 ± 1.4 ms) and CMS (2.0 ± 1.4 ms), and these responses often showed multiple peaks with the PSTH peak having a long duration (65.3% and 44.9%, respectively). Such long-duration excitatory responses with multiple peaks were rarely observed in the finger muscles following TES or in the BB following stimulation of the Ia fibers. The responses obtained with TES were compared in the same 14 BB MUs during the co-contraction and isolated BB contraction tasks. Eleven and three units, respectively, exhibited activation with multiple peaks during the two tasks. In order to determine the dispersion effects on the axon conduction velocities (CVs) and synaptic noise, a simulation study that was comparable to the TES experiments was performed with a biologically plausible neuromuscular model. When the model included the monosynaptic-pyramidal tract, multiple peaks were obtained in about 34.5% of the motoneurons (MNs). The experimental and simulation results indicated the existence of task-dependent disparate inputs from the pyramidal tract to the MNs of the upper limb. These results suggested that intercalated interneurons are present in the spinal cord and that these interneurons might be equivalent to those identified in animal experiments.

## Introduction

The primary motor cortex (M1) controls movement through groups of descending tracts (Lawrence and Kuypers, 1968a,b; Kuypers, 1981; Dum and Strick, 1996; Armand et al., 1997). Of the descending pathways, monosynaptic cortico-motoneuronal (C-M) connections appear to be unique to primates and particularly well developed in more dexterous species, including humans (Iwatsubo et al., 1990; Bortoff and Strick, 1993; Porter and Lemon, 1993; Maier et al., 1998).

In anesthetized macaque monkeys, however, non-monosynaptic excitation of the pyramidal tract, intercalated by spinal interneurons, has been observed in forelimb motoneurons (MNs) following the alleviation of glycinergic inhibition (Alstermark et al., 1999; Isa et al., 2006). This indicates that the effects of non-monosynaptic pathways could be masked by inhibitory systems in the spinal cord. Importantly, these pathways are vital for the recovery of voluntary movements in animals after spinal cord injury (SCI; Raineteau and Schwab, 2001; Sasaki et al., 2004; Thuret et al., 2006; Nishimura et al., 2007; Kinoshita et al., 2012). If this is also true for humans, investigation of the existence of non-monosynaptic C-M pathways is crucial for better understanding of the mechanisms underlying the motor recovery of spinal cord disorders, such as SCI and cervical myelopathy (Igarashi et al., 2011). However, the role of such pathways in humans remains contentious (Maier et al., 1998; Dietz, 2002).

To examine C-M excitation in humans, peristimulus time histograms (PSTHs) of the firing probability of motor units (MUs) are used after stimulation of M1 and the pyramidal tract through techniques such as transcranial magnetic stimulation (TMS), transcranial electrical stimulation (TES) and cervicomedullary stimulation (CMS; Day et al., 1989; de Noordhout et al., 1999; Petersen et al., 2002; Taylor, 2006). Using this technique, the rising time of the compound excitatory post-synaptic potentials (EPSPs) of the MN can be observed even in humans (Fetz and Gustafsson, 1983; Pierrot-Deseilligny and Burke, 2005). However, previous studies that investigated the proximal muscles (e.g., biceps brachii [BB]) with PSTHs following single TES and/or CMS have failed to clearly reveal non-monosynaptic connections during isolated target muscle contraction (de Noordhout et al., 1999; Petersen et al., 2002). Considering that inhibitory effects of the spinal cord mask the indirect C-M excitation in monkeys (Alstermark et al., 1999; Isa et al., 2006), non-monosynaptic excitation may be substantiated by a detailed examination of PSTHs during functional motor tasks. In fact, it has been reported that motor tasks involving activation of multi-joint forelimb muscles can more efficiently activate the presumed spinal interneuronal system by increasing the sensory inputs around the co-contracted muscles (Burke et al., 1992; Mazevet and Pierrot-Deseilligny, 1994). Thus, modulation of the MU firing probability in the PSTH under “more natural motor tasks” might indicate the existence of non-monosynaptic C-M pathways in humans.

Therefore, we hypothesized that analysis of the PSTH peaks following single stimulations of the pyramidal tract during functional motor tasks, such as co-contraction of proximal and hand muscles, would provide experimental evidence for non-monosynaptic C-M pathways in humans. To enhance the reliability of our findings and exclude contamination by conduction velocity (CV) dispersion from existing monosynaptic C-M connections (Kohara et al., 1999), we also conducted computer simulation experiments (Cisi and Kohn, 2008; Elias et al., 2012; Watanabe et al., 2013).

## Materials and Methods

### Human Experiments

The subjects were 22 healthy volunteers (19 men, 3 women; 20–46 years) who provided written informed consents to participate in the experiments. The ethics committee of Chiba University approved all study protocols, and all procedures used in the study conformed to the Declaration of Helsinki.

### Recordings

**Figure 1:**
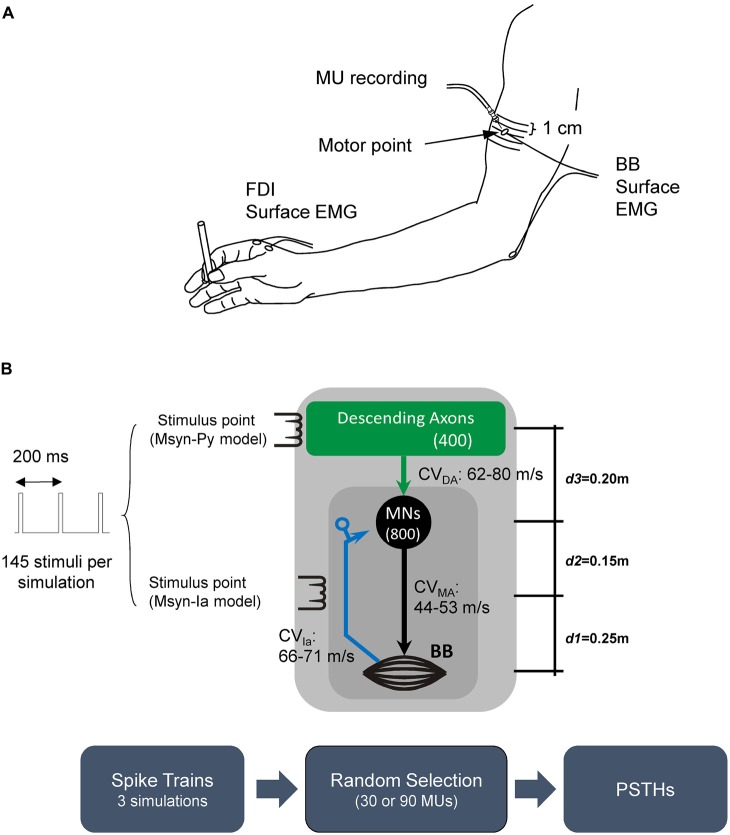
**(A)** Experimental set up and **(B)** schematic diagram of the different simulation scenarios. The Monosynaptic-Ia (Msyn-Ia) model involved activation of a monosynaptic reflex pathway by Ia afferents from muscle spindles (blue arrow and dark gray square). The descending monosynaptic pathway is indicated by the Monosynaptic-Pyramidal tract (Msyn-Py) model (green arrow and light gray square). Irrespective of the model (Msyn-Ia or Msyn-Py), 145 stimuli were delivered to a given pathway every 200 ms (stimulus rate, 5 Hz). The values (m) on the right (from bottom to top) indicate the distances between the muscle and the stimulus point (subclavicular fossa; *d1*), stimulus point and motoneuron (MN) pool (*d2*), and MN pool and scalp (*d3*), respectively. The range of the conduction velocities (CVs) for the descending axons (CV_DA_), motor axons (CV_MA_) and Ia afferents (CV_Ia_) are listed near the arrows. The box diagrams at the bottom represent the workflow for the simulation protocols. The workflow of the simulation is illustrated at the bottom.

The EMG activity of the BB and first dorsal interosseous (FDI) muscles was recorded with surface Ag-AgCl electrodes (diameter, 1 cm). In each muscle, one electrode was placed on the innervation point, which was identified by the muscle twitch with the lowest threshold (motor point; Figure 1A; Kimura, 2001). This point in the BB was usually located on the medial side of the muscle belly. Most neuromuscular junctions are thought to be located around a transverse plane crossing this point. The reference electrode was placed over the distal tendon. The surface EMG signals were amplified (1000×), band-pass filtered (15–10,000 Hz), and digitized and sampled (rate, 10 kHz) with Spike 2 software (version 6; Cambridge Electronic Design Limited, Cambridge, UK).

The activity of a single MU was recorded with bipolar needle electrodes (NM-030T; Nihon Kohden Corporation, Tokyo, Japan) that were inserted into the BB (Figure 1A). The needle was inserted so that the tip was close to the neuromuscular junction, which was located relative to the location of the innervation point. The intramuscular EMG activity was amplified (10,000×), band-pass filtered (60–10,000 Hz), and digitized and sampled at 10 kHz. In order to help the subjects maintain activity in the target MU, visual and auditory feedback activity was provided.

### Stimulation

We stimulated the corticospinal tract with electrical or magnetic stimulation of the pyramidal tract at the cervicomedullary level (CMS), TES or TMS of the M1 contralateral to the recording side. To assess the non-monosynaptic C-M excitation mediated by spinal interneurons, modulation of the PSTH (i.e., multiple peaks and long duration of PSTH peak) after a single TES would be mainly argued in this study. TES was used instead of TMS, since TMS indirectly activates pyramidal tract cells so that indirect (I) waves reflect cortical excitation (Day et al., 1989). However, the multiple peaks (e.g., late component) following TES could be caused by I waves driven by synaptic activation of pyramidal tract cells in M1 depending on the stimulus strength (e.g., high intensity; Rothwell, 1991). Therefore, we used CMS to examine whether the multiple peaks following TES accounted for the subcortical mechanisms. This stimulation could not generate I waves from the motor cortex due to CMS alone (Taylor, 2006).

To electrically stimulate the pyramidal tract, a high-voltage electrical current (duration, 100 μs; maximum output, 1 A) was applied with a DS7 stimulator (Digitimer Ltd., Hertfordshire, UK) through plate electrodes that were attached to the skin (1–2 cm posterior and superior to the tips of the mastoid processes with the cathode placed contralateral to the side of the MU recordings; Ugawa et al., 1995; Petersen et al., 2002; Taylor, 2006). For magnetic stimulation of the pyramidal tract, a Magstim 200 double-cone magnetic coil (The Magstim Company Ltd., Carmarthenshire, UK) was placed over the back of the head. The responses were best when the central section of the coil was positioned over or near the inion and the current was directed downward in the coil (Ugawa et al., 1994; Taylor, 2006). We normally used magnetic pyramidal stimulation because it tended to be more comfortable for the subjects. However, magnetic stimulation is less effective for pyramidal stimulation than electric stimulation (Taylor, 2006), and we were unable to evoke any responses in some subjects. In such cases, activation of the pyramidal tract was achieved with electrical stimulation. The compound MEP latencies were similar for both electrical and magnetic stimulation. These methods are thought to activate the axons of the pyramidal tract at almost identical points. We did not detect any differences when the two methods were compared. We therefore present the data for both methods in the present study. In addition, we determined the CMS threshold before the start of the experiments.

The M1 arm area was stimulated with either TES or TMS. TES was administered with a DS7 stimulator. The stimulating anode was placed on the scalp over the cortical area representing the target muscle. The reference cathode was located at the vertex. For TMS of the motor cortex, a figure-of-eight coil (inner diameter, 8 cm; outer diameter, 11.5 cm; Magstim 200) was used for the stimulation. The junction region was placed over the M1 arm area to induce anteromedial current flow in the brain (Sakai et al., 1997).

To investigate the mechanisms underlying excitations involving multiple peaks with long durations, we examined pyramidal tract excitation in the flexor digitorum superficialis (FDS) muscle and FDI with TES and Ia excitation in the BB with stimulation at Erb’s point. In the latter case, single-pulse electrical stimuli were delivered with the DS7 stimulator with a cathode on the supraclavicular fossa and anode on the acromion (duration, 100 μs). The stimulus intensities were adjusted so that they were just above threshold for Ia excitation, and the minimal direct motor response was concomitantly recorded by surface electrodes.

### Single MU Responses to Stimulation of the Corticospinal Tract

The subjects were seated on a reclining armchair with one of their forearms extended anteriorly and supported on a table on the ulnar side (Figure 1A). The arm was strapped to the table at the wrist. The subjects performed weak elbow flexion (0.3–3.6% of the maximum voluntary contraction [MVC]), so that one MU could be clearly distinguished in the recording (isolated contraction task). In another trial, the subjects were asked to maintain precision grips (0.5–15% MVC in the FDI) during the recordings by holding a 7-mm-diameter cylindrical object during the BB contraction (co-contraction task). Auditory and visual feedback were used to help the subjects maintain a constant rate (~10 Hz) of firing of the unit. A custom-made spike discriminator was used to generate a digital Transistor-Transistor Logic pulse in response to the activity of each MU, while the spikes were monitored with a digital oscilloscope and Spike 2 software. The stimuli were computer-controlled and delivered at 5–8-s random intervals. The stimulus intensities were adjusted so that they were just above the threshold for pyramidal excitation by TES, TMS and CMS with concomitant recording of the minimal MEPs (mean amplitude: 0.19 ± 0.14 mV) by surface EMG.

However, we sometimes used lower intensities in case stronger stimulation induced activity in other MUs that was indistinguishable from target MU activity. Therefore, we collected data on the effects that were mediated by low-threshold MUs during each recording session. Pyramidal tract stimulation was performed with different methods in separate runs, even in the same units. The data were generally compiled from the responses to approximately 100 stimuli, although 150 stimuli were used in some cases to increase response clarity.

### Analysis

The offline analysis of the unit firing was performed with the spike template-matching algorithm in the Spike 2 software and the PSTHs that were constructed with 0.1-ms bin widths (Alstermark et al., 1999; de Noordhout et al., 1999; Petersen et al., 2002). Possible false triggers were determined by visual inspection of the individual sweeps in the 80-ms period surrounding each stimulation (20 ms before and 60 ms after the stimulation). For each unit, the PSTHs were modified offline in order to show the timing of spike initiation instead of the trigger. The PSTHs from the CMS, TES and TMS experiments included averages of 93 (range, 42–122), 104 (range, 43–150) and 105 (range, 100–124) stimuli, respectively.

**Figure 2:**
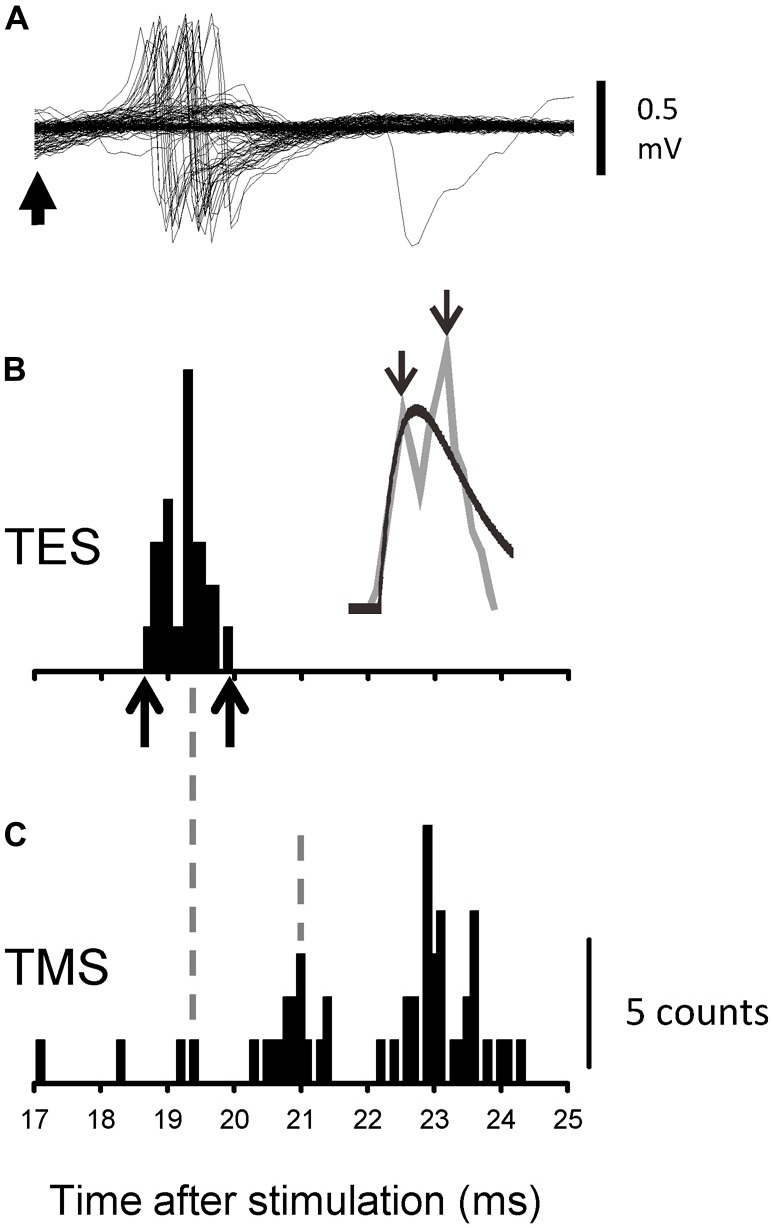
**Recordings from one representative motor unit (MU) following transcranial electrical stimulation (TES) and transcranial magnetic stimulation (TMS) during the co-contraction task. (A)** Superimposed recordings of the activity of a single MU after TES. The upward arrow indicates 17 ms after TES. **(B)** Peristimulus time histograms (PSTHs) that were created from the recordings shown in **(A)**. The identities of the spike activities were confirmed offline with a spike template-matching algorithm. The upward arrows along the abscissa indicate the onset and offset of the evoked activities. The gray line plots the moving average of the PSTH, together with the fitted curve (black line). The downward arrows indicate the peaks for which the latencies were measured. **(C)** The PSTH that was created with the recordings after TMS of the same MU as shown in **(A,B)**. The timing of some peaks is indicated by vertical broken lines.

**Figure 3:**
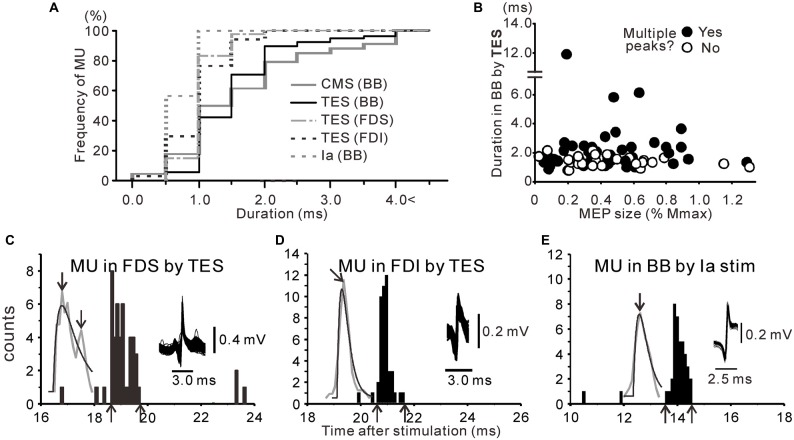
**(A)** Cumulative histograms for the duration of the excitations observed following cervicomedullary stimulation (CMS; gray line), TES (black line) and Ia stimulation (gray dashed line) in the biceps brachii (BB) muscle. The corresponding histograms for the flexor digitorum superficialis (FDS; gray dash-dot line) and first dorsal interosseous (FDI; black dashed line) after TES are also shown. **(B)** Scatter plots of the duration after TES against motor-evoked potential (MEP) size in simultaneously recorded surface electromyography (i.e., stimulus strength). The filled symbols represent responses with multiple peaks. **(C–E)** PSTHs obtained from MUs of the FDS **(C)** and FDI **(D)** after TES and BB after Ia afferent fiber stimulation **(E)**. The upward arrows indicate the onset or offset of the evoked activities, and the downward arrows indicate the measured peaks.

**Figure 4:**
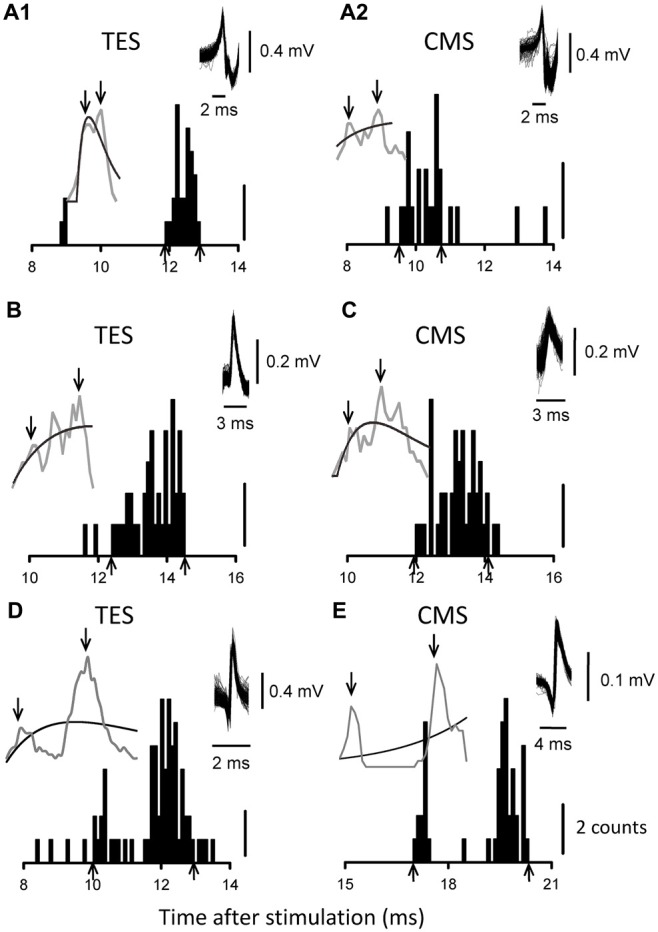
**PSTHs from five MUs.** The results obtained with TES are shown in **(A1,B,D)**, while those obtained with CMS are shown in **(A2,C,E)**. **(A1,A2)** are from the same MU. The upward arrows indicate the onset or offset of the evoked activities, and the downward arrows indicate the measured peaks.

**Figure 5:**
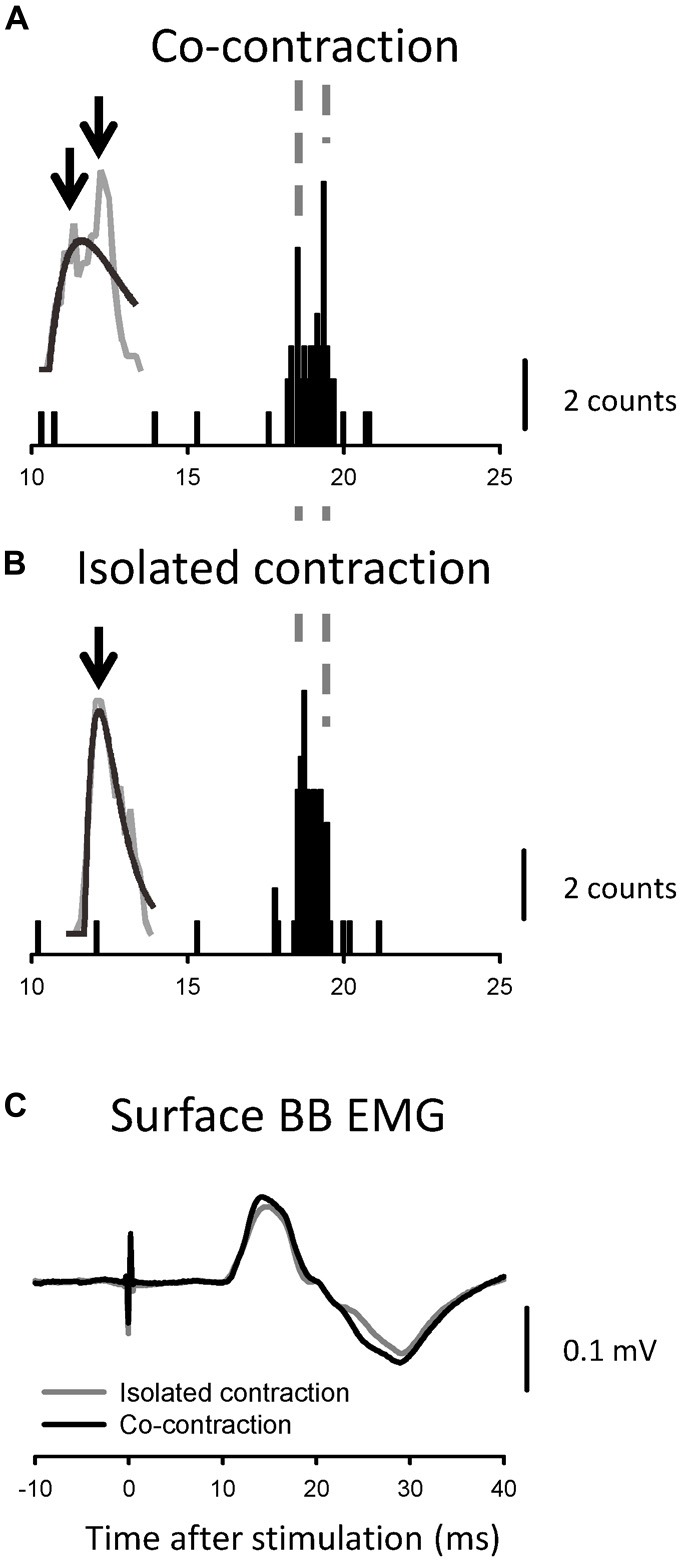
**PSTHs (A,B)** and compound MEPs **(C)** obtained from a single MU and surface electromyography that was generated by the same TES, respectively. The PSTHs that were obtained during the co-contraction task are presented in **(A)**, and those obtained during the isolated contraction task are presented in **(B)**. The timing of some peaks is indicated by vertical broken lines. The downward arrows indicate the measured peaks in the smoothed PSTH. The MEPs in the co-contraction (black line) and isolated (gray line) tasks exhibited simila sizes and shapes **(C)**.

**Figure 6:**
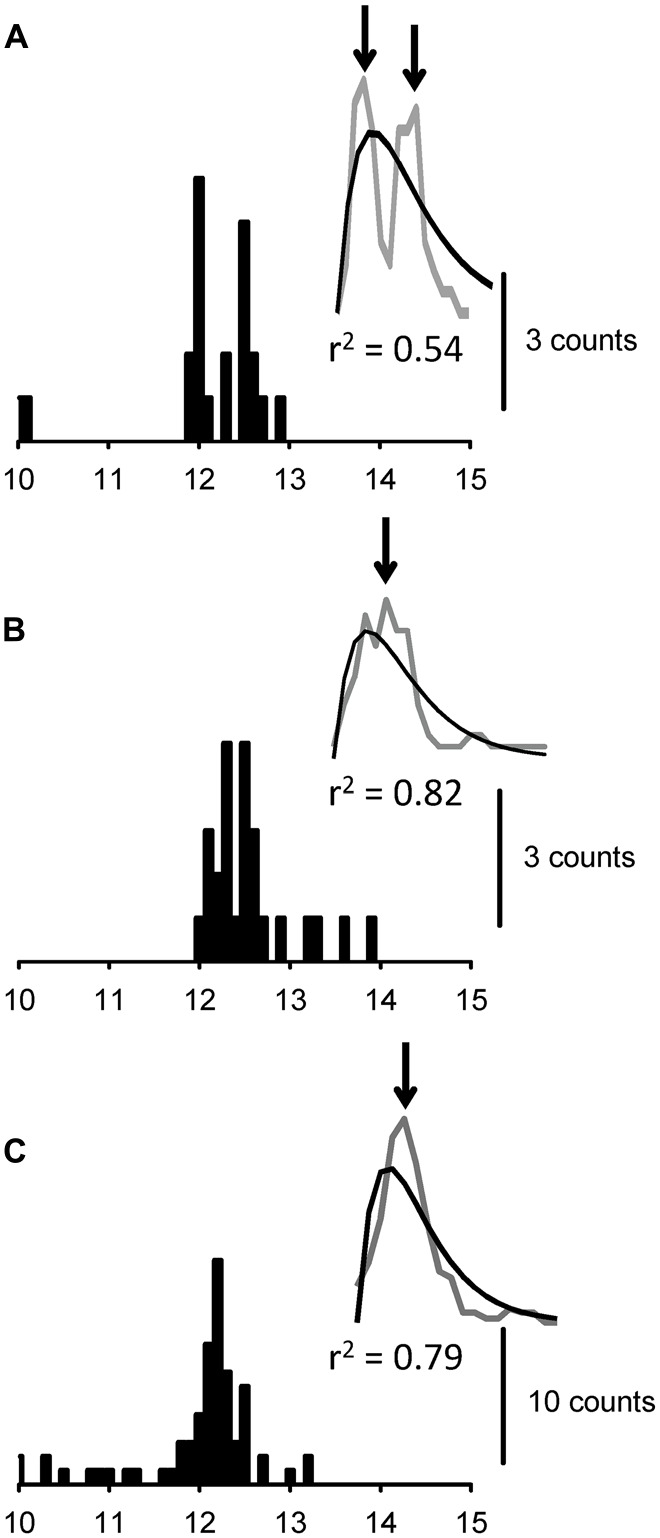
**PSTHs that were obtained with the monosynaptic pyramidal tract model and three different MUs (A–C).** The insets **(A–C)** show the moving average of the PSTH (gray curve) and the fitted curve (black curve), with their respective *r*^2^ values.

A PSTH peak of increased firing probability was detected when the following two criteria were satisfied: (1) five or more activity counts were seen in seven adjacent bins; and (2) the first two consecution of the seven bins showed spike activity. The onset of a peak was defined as the first bin. When the current conditions (background (BG) firing rate, 10 Hz; bin width, 0.1 ms; average number of trials, 100; and time window to search evoked activities, 15 ms) were considered, such activity could be observed only once out of 30 averages, at most, in the absence of evoked activity. The offset of the peak was detected with reverse application of the same criteria. The onset latency and duration of increased firing probability were measured in the PSTH. We employed a moving average (three consecutive bins) to smooth the histogram and locate any gaps (troughs) in the excitation (gray lines in Figures 2–6). In addition, we fit the smoothed histograms with spike-density functions in order to mimic simple monosynaptic EPSPs (Thompson et al., 1996) using least-squares estimations (e.g., black line over the smoothed PSTH in Figure 2B).

In the function, the firing rate [R(t)] was estimated as:

$$\text{R(t) = }{\text{[1 − exp(−t/τ}}_{\text{g}}{\text{)] × [exp(−t/τ}}_{\text{d}}\text{)],}$$

where τ_g_ is the rising-phase time constant and τ_d_ is used to adjust for the time decay.

We calculated the proportion of variance that was accounted for by the model (equivalent to *r*^2^; Statistica; Dell Statistica, Tulsa, OK, USA). Multiple components (peaks) were found if the *r*^2^ was smaller than 0.75. When the analysis suggested the existence of multiple peaks, we measured the latencies of the two peaks. In cases with more than two peaks, we only measured the first peak that was followed by a trough of more than one count, and the late peak was defined as the peak that showed the next-highest count after the first. We did not count the number of peaks because discrimination of the individual peaks was difficult in some cases. Excitations that contained multiple peaks were commonly observed with TMS, and they exhibited longer durations than the excitations obtained with the other methods. This might have been due to association of the stimulations with multiple descending volleys (Day et al., 1987; Burke et al., 1994). Consequently, we only present the data on the latency of the stimulation herein. For each unit, we also measured the voluntary recruitment threshold with respect to the MVC.

### Simulations

#### Description of the Neuromuscular Model

The MN pool model used in this study was in accordance with previously described structures (Cisi and Kohn, 2008; Elias et al., 2012; Watanabe et al., 2013). Figure 1B depicts the structure and simulation workflow used in the present study for the different two monosynaptic connections. Briefly, the MN pool encompassed two-compartment type-specified (i.e., fast-twitch fatigable [FF-type], fast-twitch fatigue-resistant [FR-type] and slow-twitch fatigue-resistant [S-type]) MN models with the geometric and electrotonic parameters that have been proposed for the lumbar MNs of anesthetized cats (Zengel et al., 1985; Cullheim et al., 1987; Fleshman et al., 1988). Here, we hypothesized that both lumbar and cervical neurons have similar dynamic behaviors.

The somatic compartment includes the ionic conductances that are responsible for the genesis of action potentials (Na^+^ and fast K^+^) as well as after-hyperpolarizations (slow K^+^). The dendritic compartment exhibits purely passive behavior because it has no active ionic channels. The time courses of each active conductance were simplified (Destexhe, 1997) in order to speed up the simulation of thousands of neuronal elements.

The MN models were parameterized in order to examine the basic dynamic behaviors that have been previously reported in studies of anesthetized cats (e.g., gain of frequency-current [*f-I*] curves, after-hyperpolarization magnitude and duration). The MN parameters were varied in a piecewise linear fashion in order to account for the range of passive properties in the MN pool.

The total number of MNs in the pool of the BB muscle has been estimated as approximately 800 (Enoka, 2008). Thus, we adopted this value to represent the BB MN pool, which is made up of 50% S-type and 50% F-type (25% FR- and 25% FF-type) MNs (Johnson et al., 1973). The synaptic conductances in the MNs were in accordance with the kinetic model proposed by Destexhe et al. (1994). For the MNs, the conductance was placed in the dendritic compartment, and the parameters were adjusted to represent the time courses and peaks of the EPSPs of anesthetized cats (Finkel and Redman, 1983).

The motor axon CVs were varied in a piecewise linear fashion with a given variability (Gaussian random variable with 10% coefficient of variation). For instance, the ranges of the mean values are 44–47 m/s for S-type MUs, 47–50 m/s for FR-type MUs and 50–53 m/s for FF-type MUs (Nardone and Schieppati, 1998; Cisi and Kohn, 2008). The sensory Ia afferent fibers were modeled as simple threshold detectors with an associated CV (66–71 m/s; Pierrot-Deseilligny and Burke, 2005). For a given Ia afferent subjected to electrical stimulation, a spike was generated only if the amplitude of the electrical stimulus that was applied to the peripheral nerve was higher than the given threshold. The range of thresholds was normalized so that the last afferent was recruited when the electrical stimulus reached the arbitrary value of 1. The total number of Ia afferents in human BB has been estimated as 320 (Banks, 2006). A 90% connectivity rate was adopted for the monosynaptic-Ia (Msyn-Ia)-MN connection (i.e., each axon innervates approximately 90% of the MNs in the pool).

Four hundred independent descending axons were used to drive the MNs. The connectivity between the descending axons and MNs was 30% for all of the simulated conditions. Although this value did not significantly influence the analysis, it was chosen in order to reduce the degree of synchronism between the MUs. The BG firing of each axon was modeled as a homogeneous Poisson point process with a mean rate that was sufficient for recruiting some MNs of the pool and activating their discharge at a rate of approximately 10 Hz, which was similar to the experimental condition. The CVs of the descending tracts were linearly distributed from 62–80 m/s in accordance with the results of a study of pyramidal tract stimulation that was conducted in humans by Ugawa et al. (1995). Another population of neurons with slow CV (~13 m/s) is known to comprise a large part of the pyramidal tract neurons in animal experiments (Takahashi, 1965; Edgley et al., 1997). However, the MEPs in neurologically intact humans have been reported to give rise to the fast conducting axons in the corticospinal tracts following low-intensity TES (Rothwell, 1991; Burke et al., 1993; Rothwell et al., 1994; Kohara et al., 1999). This evidence was confirmed by epidural recordings of the human spinal cord (Burke et al., 1993; Di Lazzaro et al., 1998). If slow conducting axons create PSTH peaks with fast axons after the stimulation, then the difference of the two PSTH peaks for the fast and slow conductance axons might be too long to explain the putative oligosynaptic range of the pyramidal tract system (Kohara et al., 1999). Thus, the range of the CVs that was based on the results of Ugawa et al. (1995) was considered reasonable for the simulation study.

In order to mimic stimulation of the descending tracts by TMS, TES or CMS, a normalized threshold was attributed to each descending axon so that the stimuli that were greater than this threshold value generated spikes in the corresponding axons.

#### Simulation Protocols

Each simulation lasted 30 s. However, the data for the first second of each session were excluded from the analysis in order to avoid any initial transient of the system. The data for the last 29 s were used to evaluate the spike trains of the discharging MUs. Irrespective of the stimulation method, above-threshold stimuli were delivered to the peripheral (Ia afferents) and descending axons with a rate equal to 5 Hz (200-ms interval) so that the data for 145 stimuli were evaluated for each MU (Figure 1B). Two simulations were run for each condition presented below. The models, which were implemented in Java^TM^ programming language (Oracle Corporation, Redwood City, CA, USA), were numerically integrated with a fixed-step fourth-order Runge–Kutta method that had a time step equal to 0.05 ms.

PSTHs were constructed for single spike trains that were randomly chosen from the pool of active MUs (bottom of Figure 1B). The sort process of a given MU involved repositioning so that the same MU could be selected again in the two simulations. However, any MUs that were selected in a previous simulation were discarded in the subsequent simulations. The number (*n*) of evaluated PSTHs differed between conditions in order to match the *n* in the human experiments. Multiple peaks were detected as described above in the “Analysis” Section with a custom Matlab^®^ program (The MathWorks, Inc., Natick, MA, USA). This same software was used to sort the MUs and construct the corresponding PSTHs.

##### The Monosynaptic-Ia (Msyn-Ia) model

This condition was used to simulate the experimental condition in which electrical stimulation was delivered to the Ia afferents of the BB. As shown in Figure 1B, the distance between the muscle (mean point) and stimulus point (subclavicular fossa) was *d1*, which was 25 cm, while that between the MN pool and the stimulus point was *d2*, which was 15 cm.

The mean interspike interval for each stochastic point process was set at 10 ms in order to provide the desired BG firing (~10 Hz) for the given proportion of MNs in the pool.

##### Monosynaptic-pyramidal tract (Msyn-Py) model

This condition was used to test the hypothesis that PSTHs with multiple peaks were obtained only by the dispersion of descending axon CVs and synaptic noise. Figure 1B shows a schematic diagram of the stimulation with the descending axons providing monosynaptic connections to the MNs within the pool. Note that the stimulus point was moved from the peripheral nerve, as in the Msyn-Ia model, to the scalp (*d3* = 20 cm), which was comparable to the TES experiment. The mean interspike interval for each stochastic point process in this condition was 5 ms. This value was lower than that adopted in the Msyn-Ia model because the CV in this condition was associated with the descending axons (see model description in the “Simulations” Section), and it contributed dead time to the stochastic point processes. In addition, the innervation ratio was lower than that in the Msyn-Ia model.

### Statistics

Linear regression analyses and *t*-tests were performed. The *χ*^2^ test was used to compare the frequencies of the multiple peaks in the experiments (BB vs. FDS or FDI, FDS vs. FDI and C-M vs. Ia excitation) and computer simulation data. In order to assess the different configurations of the excitations in the MUs, we performed the nonparametric Wald–Wolfowitz runs test on the latencies of the spike activities in a temporal window, in which the MUs showed evoked activities under the two tasks. The Wald–Wolfowitz runs test is a non-parametric test of the randomness hypothesis of a two-valued data sequence. A run of a sequence was defined as a segment that consisted of equal elements (i.e., co-contraction or isolated contraction). The null hypothesis was the runs of spike activity were similarly distributed in the two tasks. Statistical significance was set at *p* < 0.05. The population estimates are given in the text as means ± standard deviations (medians).

## Results

### Single MU Responses Following TES and TMS

Figures 2A,B show the responses of the BB MUs following TES and the calculated PSTHs for the co-contraction task for a single subject. The MU firing probability increased around 19 ms after TES during the co-contraction task, and dense regions with slight time intervals were observed (Figure 2A). The PSTHs that were constructed from these recordings clearly exhibited peaks from multiple sources (Figure 2B). In this MU, the onset latency, difference in peak latency, and total duration of excitation were 18.6 ms, 0.5 ms and 1.1 ms, respectively. For this PSTH, the proportion of variance that was accounted for by the spike density function (*r*^2^) was 0.66. Therefore, the activity of this MU was statistically detected as double peaks (see “Materials and Methods” Section). In total, we tested 75 MUs following TES during the co-contraction task. For each unit, the duration of excitation, which was determined by analyzing the respective PSTHs, was determined to be 1.9 ± 1.4 (1.6) ms (*n* = 75; black line in Figure 3A). To illustrate the variability between units, Figure 4 shows the PSTHs following TES of three other units (A1, B and D). Based on the criteria described in the Methods, 49/75 (65.3%) units exhibited multiple peaks following TES during co-contraction. In these units, the mean difference in latency between the first and late peaks was 0.84 ± 1.5 (0.42) ms. No differences in the recruitment threshold were found relative to the number of peaks (i.e., presence or absence of multiple peaks; *p* > 0.7, *t-test)*. Furthermore, interindividual variability of the muscle contractions in the BB and FDI (i.e., BG EMG activity obtained from the surface electrode) did not affect these BB PSTH results during the co-contraction task. For instance, the correlation coefficients were not significant between the BG EMG levels (FDI or BB) and duration of the BB PSTH (FDI contraction vs. BB PSTH duration: *y* = 0.00004*x* + 0.0018, *R*^2^ = 0.015, *p* > 0.2; BB contraction vs. BB PSTH duration: y = −0.00001*x* + 0.0002, *R*^2^ = 0.00003, *p* > 0.9). In addition, the differences in the BG EMG levels (FDI or BB) were not related to the number of peaks (i.e., presence or absence of multiple peaks; BB: *p* > 0.9; FDI: *p* > 0.7; *t-test*).

In order to clearly show the differences in the PSTH configurations according to TES or TMS, a PSTH was constructed following TMS for the same MU shown in Figures 2A–C. The intensities of the TMS and TES were 1.08 times the active motor threshold (aMT) and 1.15× the aMT, respectively. Although a couple of peaks can be seen in the PSTH following TMS, the peak latency of the earliest component was not the same as that of the late component in the PSTH following TES (see Figures 2B,C). Next, we used TMS to test 44 of the 49 MUs that exhibited multiple peaks with TES. The peak latencies of the first component in 40 of the MUs (91%) with TMS were longer than those of the late component with TES. In addition, the generation of multiple peaks by TES was less related to the size of the MEP (i.e., stimulus strength) that was simultaneously recorded by the surface EMG (Figure 3B).

Analyses of the existence of multiple peaks in the distal muscles following TES or in the BB after Ia stimulation in the PSTHs were also conducted. As shown in Figure 3, excitations with multiple peaks were seen in the FDS (Figure 3C, *n* = 41), but they were indiscernible in the FDI (Figure 3D, *n* = 35), even during the co-contraction task following TES. In addition, the same was true following Ia stimulation, even in the BB (Figure 3E).

To compare the PSTH configurations following TES in the different muscles and Ia stimulation in the BB, we created cumulative histograms of the PSTH peak durations that were obtained from all of the MUs. In the BB (Figure 3A), the cumulative curve exhibited a function that increased rather slowly, and the mean duration of the PSTH peaks was ~1.9 ms following TES (black line). In contrast, the curves for the FDS, FDI, and Ia stimulation (BB) exhibited functions that sharply increased due to the relatively short durations of the PSTH peaks, and the mean durations were 1.3 ± 0.3 (1.3), 1.2 ± 0.4 (1.1) and 0.9 ± 0.2 (0.9) ms, respectively. As a result, the durations of the PSTH peaks were significantly shorter for both the FDS and FDI compared with that for the BB (*p* < 0.01, *t*-test). Furthermore, the frequency of the multiple peaks was significantly lower only in the FDI (20.0%) compared to that of the BB (65.3%, *p* < 0.001, *χ*^2^ test) during co-contraction. A significant difference in frequency was also seen between the FDS (61%) and FDI (*p* < 0.001, *χ*^2^ test).

### CMS

In order to examine whether the multiple peaks that were induced by TES accounted for the subcortical mechanisms, we used CMS to examine pyramidal tract excitation. Figures 4A2,C,E show PSTHs following magnetic CMS of three MUs. In total, we tested 56 and 13 MUs with magnetic and electrical CMS, respectively. Of the 69 units, we also assessed the responses induced by TMS in 59 units and by TES in 19 units. The mean duration of the excitation induced by CMS (either electrical or magnetic) was 2.0 ± 1.4 (1.4) ms (*n* = 69), which was very similar to the duration induced by TES, as described above (gray solid line in Figure 3A).

We examined whether the multiple peaks in the excitations were induced by both electrical and magnetic CMS during the co-contraction task. Multiple peaks were seen in 31/69 (44.9%) units, which was a slightly smaller frequency than that induced by TES. This result might be partly explained by the shorter conduction distances in CMS compared to those in TES, although almost half of the units still showed multiple peaks. Of these, 11 units were also tested by TES, and eight units exhibited multiple peaks with both methods (Figures 4A1,A2). The mean difference in latency between the first and late peaks in these units was 1.26 ± 0.91 (0.94) ms. In summary, the responses obtained with electrical and magnetic CMS closely resembled those obtained with TES. The number of peaks (presence or absence of multiple peaks; *p* > 0.9, *t*-test) and total duration (*r*^2^ = 0.0002, *p* > 0.05) were not influenced by the recruitment thresholds of the MUs.

### Effects of the Tasks on Pyramidal Tract Excitation

As described above, we frequently observed pyramidal tract excitations with multiple peaks. In previous studies conducted on humans, pyramidal tract excitation in the BB exhibited single peaks with short durations in the PSTHs that were obtained with TES or CMS (de Noordhout et al., 1999; Petersen et al., 2002). In order to investigate whether this difference was due to the subjects performing the task during the recordings, we examined corticospinal excitation during the different tasks. Figures 5A,B shows the PSTHs for one MU in the BB during the two tasks (i.e., contraction of the BB with [A: co-contraction] and without [B: isolated contraction] a precision grip). The unit showed two peaks during co-contraction (*r*^2^ = 0.49; Figure 5A). However, the same MU predominantly showed a single peak during isolated contraction (*r*^2^ = 0.89; Figure 5B), and these configurations were significantly different (*p* < 0.05, runs test). In this unit, the stimulus strengths were 1.06 times the aMT during both co-contraction and isolated contraction. The MEPs displayed similar sizes and shapes during the two tasks (Figure 5C).

In total, we examined 14 MUs during the two different tasks and found that 11 units showed multiple peaks during co-contraction (*p* = 0.008, *χ*^2^ test). The mean duration of excitation was 2.0 ± 0.9 (1.8) ms during co-contraction. Conversely, only four units showed multiple peaks during isolated contraction. Therefore, the mean duration of excitation decreased slightly during the isolated contraction task (1.7 ± 0.5 [1.6] ms), with one unit showing a decrease over 1 ms.

### Simulation Results

Representative PSTHs for the three MUs in the Msyn-Py model (see “Simulation Protocols” Section above), along with the moving average and Thompson’s fit, are shown in Figure 6. This condition showed single or multiple peaks, depending on the MU selected. Thus, PSTHs with multiple peaks were obtained, even in the monosynaptic model (Figure 6A). For a subset of ~30 MUs, we found multiple peaks for 10.7% (3/28) and 34.5% (10/29) of the MUs in the Msyn-Ia and Msyn-Py models, respectively. These different percentages (*p* = 0.033, *χ*^2^ test) suggested that the dispersion of CVs, which was higher in the Msyn-Py model, was a factor that affected the occurrence of multiple peaks in the PSTHs. The descending monosynaptic connection model was re-evaluated in a relatively large number of MUs, and multiple peaks were found in 37.0% (30/81). The difference in the latencies between the peaks was 0.39 ± 0.14 ms.

## Discussion

In the present study, we demonstrated that the performance of a functional motor task (i.e., co-contraction task of the BB and hand muscles) yielded multiple peaks in the PSTHs following pyramidal tract stimulation (TES and CMS). Moreover, the proportion of multiple peaks clearly increased during the co-contraction task compared to those during the isolated BB contraction. In addition, the durations of the pyramidal tract excitation in our study were 1.9 and 2.0 ms after TES and CMS, respectively, during the co-contraction task. These findings were replicated by the simulation experiments, which suggested that the multiple peaks in the PSTHs following the pyramidal tract stimuli cannot solely be explained by dispersion of the CVs of the descending tracts. Previous studies have shown that single TES and CMS produced a single peak with a short duration (1.1 ms for TES and 1.3 ms for CMS) in the PSTH, indicating a dominant monosynaptic component of the BB (de Noordhout et al., 1999; Petersen et al., 2002). Similar to our study, both studies (de Noordhout et al., 1999; Petersen et al., 2002) used the same bin width (0.1 ms) and number of stimulations to construct the PSTHs (*n* = 100), except that an isolated contraction task was used during pyramidal tract stimulation (de Noordhout et al., 1999; Petersen et al., 2002). Thus, the differences in the motor tasks might have contributed to the discrepancies among the results of the studies.

### The Underlying Mechanisms of Excitations with Multiple Peaks and Long Durations

Several mechanisms might underlie the multiple peaks of the excitations. First, this phenomenon could be explained by the longitudinal distribution of multiple neuromuscular junctions over several centimeters in BB muscle fibers (Masuda et al., 1983; Aquilonius et al., 1984). Because the CV of muscle fiber is very slow (1.1–12.5 m/s; Li and Sakamoto, 1996), remote innervation could induce multiple peaks from a single stimulation. However, this is unlikely because muscle fibers with double innervation are rare in adult muscles, particularly in the BB (Li and Sakamoto, 1996; Lateva et al., 2002, 2003).

The second possibility is that excitations with multiple peaks (e.g., late component) following TES are caused by I waves that are generated by synaptic inputs to pyramidal cells in M1. Since anodal electrical stimulation (i.e., TES) over the human motor cortex generally activates the axon hillock or a site just proximal to the axons of pyramidal tract neurons, a modulation of C-M excitation (D wave) can ascribe subcortical mechanisms (Rothwell, 1991). Indeed, in 91% of the MUs that were recorded with multiple peaks the latency of the first component of the PSTH that was induced by TMS was longer than that of the late component induced by TES (see Figures 2A,B). In the present study, the coil used for TMS was placed over the M1 arm area in order to induce anteromedial current flow in the brain, and this orientation has been reported to mainly elicit early I-wave components in humans (Werhahn et al., 1994; Sakai et al., 1997). Furthermore, the strengths of both stimulations (TES: 1.07 ± 0.09 times aMT, TMS: 1.2 ± 0.15 times aMT) were quite low. Di Lazzaro et al. (1998) demonstrated that strengths that were 1.5 and 2.0 times aMT were needed in the surface EMGs to evoke early I waves with TES (i.e., I1) and D waves with TMS, respectively, in human epidural recordings in conscious subjects. Considering the range of stimulus strengths in our study, the contributions of TES-induced I waves and TMS-induced D waves to the PSTH peaks seemed small. In summary, the late components with multiple peaks that were induced by TES were likely distinct from the peaks of the I waves that were observed in the current experiment. In addition, we found that CMS elicited multiple peaks in PSTHs without I waves in many cases. However, this potential explanation does not account for all of the late excitation that was induced by TES.

In MUs exhibiting multiple peaks, the mean interval between two peaks was 0.84 ms for TES and 1.26 ms for CMS. Thus, the short interval also seemed to exclude the possibility that the late peak was attributable to polysynaptic effects, judging from the effects of the D- and I-wave excitations of the pyramidal cells in M1 (interval, 1–2 ms; Burke et al., 1993). In other words, the interval might exclude the possibility that indirect pathways, which are intercalated by interneurons, mediate the late peak. However, the time difference between the two peaks corresponds well with the duration of monosynaptic C-M excitation in monkeys. Recordings from single MUs yield PSTH peaks with durations of 0.74 ± 0.25 ms (Olivier et al., 2001), and recordings from the soma of single MNs reveal EPSPs with a mean rise time of 0.93 ± 0.18 ms (Maier et al., 1998). The latency difference between the earliest mono- and di-synaptic pyramidal EPSPs has been reported as 0.6 ms in monkey upper-limb MNs (Alstermark et al., 1999; Sasaki et al., 2004). These findings suggest that the late peak resulted from polysynaptic pyramidal inputs to the MNs and that the polysynaptic pyramidal inputs show faster rise times than the monosynaptic pyramidal inputs. Furthermore, it is worth noting that such long-duration excitations with multiple peaks in the PSTHs have rarely been observed in distal hand muscles following TES. Because the activation of monosynaptic pathways in hand muscles is preferentially affected in PSTHs, as previously described (Palmer and Ashby, 1992; Pierrot-Deseilligny, 1996), the late peak in proximal muscles might result from factors other than the putative monosynaptic contributions to MNs.

The third possibility is that the small EPSPs that were induced by the pyramidal tract inputs on the MNs produced PSTHs that were broader than those induced by large EPSPs if synaptic noise existed in the MNs (Fetz and Gustafsson, 1983). However, this possibility seemed rather unlikely to explain our findings. In the present study, we confirmed that the BG firing frequencies and MU activity fluctuations (~10 Hz) were almost the same, even in the different tasks. Consequently, the presumed synaptic noise on the target MNs might have been relatively stable across different MU recordings. In these situations, we found that the generation of multiple PSTH peaks in the BB was unrelated to the degree of TES strength in all of the MU recordings (see Figure 3B). Thus, the multiple peaks on the PSTHs were not relevant to the amount of descending pyramidal tract excitation. For the task-dependent generation of multiple peaks, we found that the percentage was significantly higher during co-contraction tasks than during isolated contractions, even with the same intensity of TES and similarly sized compound MEPs in the two tasks (see Figure 5). This observation suggested that the task difference played a key role in generating multiple peaks. Taken together, these results suggested that the contribution of synaptic noise in the MNs to the generation of multiple peaks in the PSTHs following TES was relatively small.

Finally, we need to consider the CV dispersion in the descending fibers. Multiple peaks can be generated with appropriate time intervals due to the dispersion of CV in the descending axons and stochastic synaptic noise in the MNs’ membranes (Calvin and Stevens, 1968; Fetz and Gustafsson, 1983). However, a precise determination of the contribution of monosynaptic effects to the occurrence of multiple peaks after TES is not possible. This possibility will be discussed further in the next section.

### Simulation Data

PSTHs with multiple peaks exhibit the effects of monosynaptic inputs to a certain extent because of CV dispersion and stochastic synaptic noise. If the probability of multiple peaks resulting from monosynaptic pathways was high enough to diminish the indirect effects of the interneuronal systems, our findings would have little meaning for human motor neurophysiology. Therefore, we conducted simulation experiments in order to elucidate the synaptic effects of monosynaptic inputs on MNs, with a number of assumptions that were based on known spinal cord physiology and the results of previous studies (Cisi and Kohn, 2008; Elias et al., 2012; Watanabe et al., 2013).

In some cases, the simpler monosynaptic representation model confirmed a certain percentage of multiple peaks in the PSTHs, as expected. Thus, these simulated results suggested that the percentage of multiple peaks that were induced by pyramidal tract stimulation in the experiments conducted on humans was partly associated with the dispersion of spikes from the descending tracts to the MNs. Interestingly, the percentage of multiple peaks that was induced with this simulation (34.5%) was very close to the percentage obtained in the experiment involving isolated BB contractions (36.7%).

The percentage of multiple peaks during co-contraction was much higher (65.3%) than that obtained in the simulation model (34.5%). Assuming that the simulated results roughly represent real spinal cord neurophysiology, the difference in the percentages of multiple peaks (~30%) between the TES human experiments and monosynaptic simulation cannot be fully explained by descending tract dispersion or MN membrane potential randomness (i.e., synaptic noise; Calvin and Stevens, 1968). These findings suggested that an additional neural system (i.e., an indirect pathway from M1 to MNs) that impinged on the BB MNs played a critical role in generating ~30% of the multiple peaks. The percentage was lower in CMS human experiments (~40%), possibly because of the shorter conduction distances, which reduced the conduction dispersion.

### Interneuron Candidates for the Non-Monosynaptic Pathways from M1 to the MNs

Colebatch et al. (1990) have reported that TMS, and occasionally TES, cause medium-latency excitation in shoulder muscles in addition to early (monosynaptic) effects. Those authors also suggested that an indirect polysynaptic route caused the excitation; however, they could not exclude the effects of small-diameter corticospinal fibers following trans-synaptic excitation. In the current study, indirect pathways from M1 to the MNs were the most likely cause of the task-dependency of the excitation, which suggested that a particular task decreased the threshold of the activation of the indirect pathway. Indeed, parts of pathways are more easily activated by the co-contraction of remote muscles and the sensory inputs around them (Burke et al., 1992; Mazevet and Pierrot-Deseilligny, 1994).

Several intercalated interneurons might be involved in the indirect pathways from M1 to the MNs. One source of these neurons is the brainstem, which receives inputs from corticobulbar axons. Neurons situated here send outputs through reticulospinal fibers, for example. Another possible source is spinal interneurons, which include propriospinal neurons and segmental interneurons that have their cell bodies in segments that are remote from, as well as within, the same segments as upper-limb MNs (Isa and Nishimura, 2014). Our observations of similarly delayed excitations after both CMS and TES suggested that the delays resulted from interneuronal relays in the spinal cord. However, we could not exclude the possibility that CMS stimulated other descending and/or ascending fibers together with the pyramidal fibers (Taylor, 2006). The differences between TES and CMS shown in Figure 4 suggested the activation of various fibers, while their general similarity indicated that the fast pyramidal fibers were predominantly activated.

As discussed earlier, one possible source of spinal interneurons is the C3–C4 propriospinal system, which has been extensively studied in the cat and which has been shown to transmit corticospinal excitation to forelimb MNs (Illert et al., 1978; Alstermark and Lundberg, 1992). However, the effects mediated by indirect corticospinal projections are difficult to observe in primates. These neuronal effects have been clearly observed in macaque monkeys only after injections of strychnine, probably because of feed-forward inhibition by the intercalated interneurons (Maier et al., 1997; Alstermark et al., 1999; Olivier et al., 2001). Therefore, our relative ease of obtaining effects in the normal human subjects in the present study was puzzling. However, the monkeys examined in the previous experiments were anesthetized or sedated, while the human subjects in the present study were fully conscious, and their nervous systems were more active than the monkeys’ were. In addition, our subjects maintained the contraction of the target muscle (BB), and this might have specifically activated the pathways to the muscle. These factors might have made the indirect pathways more susceptible to pyramidal stimulation.

Evidence exists that a C3–C4 system mediated the observed effects. First, we observed long-duration pyramidal tract excitation with multiple peaks more easily with the co-contraction task, which activated the C3–C4 systems (Burke et al., 1992; Mazevet and Pierrot-Deseilligny, 1994). Second, we often observed excitations with multiple peaks in the BB and FDS but not in the FDI. Pierrot-Deseilligny (1996) noted that the effects of the C3–C4 system could be observed in various muscles, including the BB and FDS, of the human upper limb, with the exception of intrinsic hand muscles (e.g., FDI).

## Conclusion

In the present study, we observed multiple short-latency peaks in the PSTHs following TES and CMS while the subjects performed co-contraction of their forearm muscles. These findings were well replicated by the recently developed simulation experiments, which indicated that the multiple peaks in the PSTHs following pyramidal tract stimulation cannot be explained by descending tract dispersion or MN membrane potential randomness. These new findings suggest that a particular motor task generates excitation of a non-monosynaptic C-M pathway, presumably through spinal interneurons, even though there exists a predominant monosynaptic C-M pathway. Interestingly, this indirect pathway has been considered vital for the recovery of voluntary movements in animals after SCI (Raineteau and Schwab, 2001; Sasaki et al., 2004; Thuret et al., 2006; Nishimura et al., 2007; Kinoshita et al., 2012). Thus, our assessment of the existence of this pathway in humans may have potential implications for the enhancement of motor recovery through indirect C-M pathways in spinal cord disorders, such as SCI and cervical myelopathy (see Igarashi et al., 2011). However, additional studies are needed to better understand the limitations of this enhancement of motor recovery to clinical applications.

## Author Contributions

TN, TT, LAE, RAM, TK, YO conceived and designed the experiments, contributed reagents/materials/analysis tools and wrote the article. TN, TT, MS, TE, SS, LAE, RAM, TK, YO performed the experiments. TN, TT, MS, SS, LAE, RAM analyzed the data.

## Funding

This human study was supported by the following grants from the Ministry of Education, Culture, Sports, Science and Technology (MEXT)/Japan Society for the Promotion of Science (JSPS) KAKENHI: 25702033 and 26560282 (TN), 23500617 and 26120002 (YO). The simulation study was supported by Fundação de Amparo à Pesquisa do Estado de São Paulo (FAPESP), a Brazilian Funding Agency (proc. no. 2009/15802-0; 2013/10433-1; LAE) and the Bolsa Estágio de Pesquisa no Exterior (BEPE) program of FAPESP (proc. no. 2012/05304-5; RAM).

## Conflict of Interest Statement

The authors declare that the research was conducted in the absence of any commercial or financial relationships that could be construed as a potential conflict of interest.
